# Serum and Tissue Expression Levels of Leptin and Leptin Receptor Are Putative Markers of Specific Feline Mammary Carcinoma Subtypes

**DOI:** 10.3389/fvets.2021.625147

**Published:** 2021-02-10

**Authors:** Andreia Gameiro, Catarina Nascimento, Ana Catarina Urbano, Jorge Correia, Fernando Ferreira

**Affiliations:** CIISA - Centro de Investigação Interdisciplinar em Sanidade Animal, Faculdade de Medicina Veterinária da Universidade de Lisboa, Lisbon, Portugal

**Keywords:** feline mammary carcinoma, leptin, leptin receptor, free leptin index, biomarkers

## Abstract

Obesity is an established risk factor for breast cancer in post-menopausal women, being associated with elevated serum levels of leptin. Although overweight is a common condition in cat, the role of leptin and its receptor in feline mammary carcinoma remains unsettled. In this study, serum leptin and leptin receptor (ObR) levels were investigated in 58 cats with mammary carcinoma and compared with those of healthy animals, as were the expression levels of leptin and ObR in tumor tissues. The results showed that the Free Leptin Index is significantly decreased in cats with mammary carcinoma (*p* = 0.0006), particularly in those with luminal B and HER2-positive tumors, and that these animals also present significantly lower serum leptin levels (*p* < 0.0001 and *p* < 0.005, respectively). Interestingly, ulcerating tumors (*p* = 0.0005) and shorter disease-free survival (*p* = 0.0217) were associated to serum leptin levels above 4.17 pg/mL. In contrast, elevated serum ObR levels were found in all cats with mammary carcinoma (*p* < 0.0001), with levels above 16.89 ng/mL being associated with smaller tumors (*p* = 0.0118), estrogen receptor negative status (*p* = 0.0291) and increased serum levels of CTLA-4 (*p* = 0.0056), TNF-α (*p* = 0.0025), PD-1 (*p* = 0.0023), and PD-L1 (*p* = 0.0002). In tumor samples, leptin is overexpressed in luminal B and triple-negative carcinomas (*p* = 0.0046), whereas ObR is found to be overexpressed in luminal B tumors (*p* = 0.0425). Altogether, our results support the hypothesis that serum levels of leptin and ObR can be used as biomarkers of specific feline mammary carcinoma subtypes, and suggests the use of leptin antagonists as a therapeutic tool, reinforcing the utility of the cat as a cancer model.

## Introduction

Feline mammary carcinoma (FMC) is a high prevalence disease (12–40% of all tumors in cat) that shows similar clinicopathological and genetic features (1), comparing to human breast cancer (2), supporting its use in comparative oncology studies (3, 4), and allowing to improve therapeutic protocols for women and cats (5). Despite the cat is considered a suitable cancer model, especially for the most aggressive mammary carcinomas subtypes, HER2-positve (2, 6) and triple-negative (6–8), further efforts are needed to track disease progression (9). Some common biomarkers are already identified, as for example, the androgen receptor (1), the PD-1 (10) and the CTLA-4 (11), which represent potential molecular therapeutic targets. Likewise, obesity is a common nutritional disorder in the cat, with higher prevalence in indoor and sterilized animals above 3 years of age (12). In humans, obesity induces a chronic inflammatory status, being a risk factor for breast cancer (13–15).

Leptin is a 16 kDa adipocytokine, encoded by the *obese* gene and involved in the central regulation of food intake, energy homeostasis, modulation of reproductive function and peripheral metabolic processes, such as breast/mammary gland development, cellular proliferation and angiogenesis (16–18). In tissues and serum, leptin expression is modulated by fat mass, with healthy cats showing lower serum leptin levels than obese animals (12), as reported in humans (13, 14). Interestingly, although this protein is mainly secreted by adipocytes, it can also be expressed by pathologically altered cells, such as cancer cells (19, 20). Thus, malignant cells can regulate their metabolic activities (21), promoting uncontrolled cell growth via Wnt/β-catenin (22), migration, invasion and angiogenesis (15, 23), and downregulating apoptosis through a Bcl-2-dependent mechanism (21, 24). Accordingly, leptin overexpression is detected in breast cancer cells and neighboring adipocytes, contrasting with normal breast glandular epithelial cells (15, 25), promoting the expression of several tissue factors (26), which suggest an oncogenic role for this adipocytokine (14). Furthermore, studies in human breast cancer patients showed that leptin overexpression has paracrine effects, not always reflected in serum levels, but associated with more aggressive tumors and therapy resistance (25). Additionally, in overweight human patients a positive correlation was found between leptin overexpression in the tumor microenvironment and estrogen receptor (ER) positive breast cancer, and with a human epidermal growth factor receptor 2 (HER 2)-positive status frequently related to a more invasive tumor phenotype (27).

In parallel, the leptin receptor (ObR, 150–190 kDa) was found to be involved in innate and adaptive immunity (28), being expressed in several organs, including breast and peripheral tissues, as well as in adipocytes (29, 30) and immune cells. ObR has an extracellular N-terminus domain, a transmembrane domain and a cytoplasmic C-terminus domain. Upon leptin ligation, ObR homodimerizes and the associated JAK monomer is auto phosphorylated to activate the downstream signaling pathways (19); in the case of ObR forms with lack of auto phosphorylation capabilities, auxiliary kinases are important (29). The soluble ObR form is a 146 kDa protein (31) that can be generated by cellular apoptosis or by the proteolytic cleavage of the extracellular anchored protein domain, with this shedding being more frequent in shorter intracellular isoforms. In serum, ObR modulates the leptin bioavailability, being decreased in obese humans (19). In breast cancer patients, ObR is overexpressed independently of the ER status (14), being correlated with low overall survival (OS) (20). Furthermore, the ratio between leptin/ObR serum levels (Free Leptin Index—FLI) is considered a useful predictor of leptin activity, reflecting the individual metabolic status (32) and when increased it is an important risk factor for breast cancer development (33). In parallel, studies in breast cancer patients found an association between leptin and ObR overexpression with a chronic inflammatory status, conditioning T-cell immune responses (increase Th1- and decrease Th2-responses) (34) and the activation of immune checkpoint inhibitors (29). Indeed, some studies in humans have shown a positive correlation between overexpression of leptin and ObR with several immunomodulatory molecules (e.g., Cytotoxic T-Lymphocyte Associated Protein 4–CTLA-4; Tumor Necrosis Factor α-TNF-α; Programmed Cell Death-1–PD-1 and Programmed Cell Death-ligand 1–PD-L1) (35, 36). While CTLA-4 is a protein related to the inflammatory response that is increased in breast cancer patients, contributing to immune downregulation (37), TNF-α is a pro-inflammatory cytokine that induces apoptosis promoted by the absence of leptin (38). Moreover, the overexpression of PD-1 in T-cells is associated with ObR overexpression in humans with distinct tumor types (39), induced through the AKT pathway activation by oestrogens (40) and is responsible for the PD-1 mediated T-cell dysfunction (41).

As mentioned above, obesity is associated with increased leptin levels, which induces resistance to chemotherapy (42, 43). Therefore, the leptin/ObR axis has been widely studied (44) as a target for an adjuvant therapy, not only in ER-positive tumor status (42), but also in triple-negative tumors (45), in which the lack of hormonal receptors reduces the therapeutic options. Nowadays, different therapeutic strategies targeting the leptin/ObR axis are being used, namely leptin antagonists, that downregulate the leptin downstream pathways (e.g., Wnt and STAT3) (42, 44–46), leptin and ObR specific monoclonal antibodies or nanoparticles, that prevent leptin/ObR binding and, finally, soluble ObR molecules that enclose plasmatic leptin, regulating its availability (46).

To the best of our knowledge, this study is the first to evaluate the serum leptin and ObR levels, as well as tumor tissue expression of leptin and ObR in cats with mammary carcinoma. Thus, the main goals of this study were to: (1) compare the serum leptin and ObR levels of cats with mammary carcinoma stratified by molecular subtype with those of healthy animals; (2) investigate the leptin and ObR expression in tumor tissues and compare it with normal mammary tissues; (3) search for statistical associations between serum leptin/ObR levels and leptin/ObR IHC scores in tumor mammary tissues; and (4) test for statistical associations between serum leptin/ObR levels and clinicopathological features, in order to evaluate the utility of leptin and ObR as diagnostic and/or prognosis biomarkers or promising drug targets in cats with mammary carcinoma.

## Materials and Methods

### Animal Population

Paired tumor and serum samples were collected from 58 female cats, with fully documented history of FMC, exhibiting a mean age at diagnosis of 11.5 years (range 6.5–18 years), with the majority showing an undifferentiated breed and presenting an average body condition score (1–9) of 3.73 (ranging between 1 and 7). Also, 24 serum samples from healthy cats presented for elective ovariohysterectomy showing a mean age of 1.37 years (range 0.5–5.5 years) and an average body condition score (1–9) of 5.0 (ranging between 4 and 6), were collected at the Teaching Hospital of the Faculty of Veterinary Medicine, University of Lisbon. All the procedures involving manipulation of animals were consented by the owners. For each animal enrolled in the study, the clinicopathological data were recorded, including: age; breed; body weight; reproductive; and contraceptive administration status; treatment status (none, mastectomy or mastectomy plus chemotherapy); number, location, size, and histopathological classification; ER status, PR status, HER2 status (47), and Ki-67 index (48) of tumor lesions; malignancy grade, scored using the Elston and Ellis system (49); presence of tumor necrosis, lymphatic invasion, lymphocytic infiltration, and/or cutaneous ulceration; regional lymph node involvement; and clinical stage (TNM system) (Table 1). Regarding the molecular subtyping of feline mammary carcinomas (2, 50), animals were stratified in luminal A (*n* = 10), luminal B (*n* = 17), HER2-positive (*n* = 15) and triple-negative (*n* = 16) groups. The animals were anesthetized before surgical procedures and blood samples were collected without interfering with the animals' well-being. Briefly, all tissue samples were embedded in paraffin after fixation in 10% buffered neutralized formalin (pH 7.2), during 24–48 h, while serum samples were separated from clotted blood by centrifugation (1,500 g, 10 min, 4°C) and stored at −80°C until further use. All samples that showed haemolysis were discarded, as recommended (2, 51).

**Table 1:** Clinicopathological features of the female cats with mammary carcinomas enrolled in this study.

**Clinicopathological feature**	**Number of animals (%)**	**Clinicopathological feature**	**Number of animals (%)**
**Breed**		**Size**	
Undifferentiated	44 (75.9%)	<2 cm	22 (37.9%)
Siamese	7 (12.1%)	≥2 cm	36 (62.1%)
Persian	5 (8.6%)	**Animal weight**; 23 unknown	
Norwegian Forest	2 (3.4%)	<3 kg	6 (10.3%)
**Age**		3–5 kg	24 (41.4%)
<8 years old	4 (6.9%)	>5 kg	5 (8.6%)
≥8 years old	54 (93.1%)	**Treatment**; 3 unknown	
**Reproductive status**; 1 unknown		Mastectomy	49 (84.5%)
Spayed	20 (34.5%)	Mastectomy + Chemo	4 (6.9%)
Pill	21 (36.2%)	None	2 (3.4%)
Both	9 (15.5%)	**Multiple tumors**	
Any	7 (12.1%)	Yes	35 (60.3%)
**Lymph node status**; 4 unknown		No	23 (39.7%)
Positive	19 (32.8%)	**Malignancy grade**; 1 unknown	
Negative	35 (60.3%)	I	3 (5.2%)
**Stage (TNM)**		II	8 (13.8%)
I	15 (25.9%)	III	46 (79.3%)
II	6 (10.3%)	**Necrosis**	
III	31 (53.4%)	Yes	42 (72.4%)
IV	6 (10.3%)	No	16 (27.6%)
**Lymphatic invasion**		**Lymphocytic infiltration**; 2 unknown	
Yes	7 (12.1%)	Yes	37 (63.8%)
No	51 (87.9%)	No	19 (32.8%)
**HER2 status**		**Tumor ulceration**	
Positive	14 (24.1%)	Yes	8 (13.8%)
Negative	44 (75.9%)	No	50 (86.2%)
**ER status**		**Ki67 index**; 1 unknown	
Positive	31 (53.4%)	Low (<14%)	18 (31%)
Negative	27 (46.6%)	High (≥ 14%)	39 (67.2%)
**PR status**			
Positive	36 (62.1%)		
Negative	22 (37.9%)		

*n = 58; TNM, Tumor, Node, Metastasis; ER, Estrogen Receptor; PR, Progesterone Receptor*.

### Measurement of Serum Leptin and ObR Levels

The serum levels of leptin and ObR, CTLA-4, TNF-α (11), PD-1 and PD-L1 (10) were quantified by using commercial ELISA-based kits (R&D Systems, Minneapolis, USA; DY398-05, DY389, DY476, DY2586, DY1086, and DY156, respectively). For each assay, a standard curve was plotted using 10-fold serial dilutions of the recombinant proteins provided by the manufacturer, and the *r*^2^ values were calculated using a quadratic regression [*r*^2^ = 0.9976 for leptin, *r*^2^ = 0.9632 for ObR, *r*^2^ = 0.99 for PD-1, and *r*^2^ = 0.96 for PD-L1 (10)], whereas serum CTLA-4 and TNF-α concentrations were determined by using a curve-fitting equation (*r*^2^ > 0.99), as previously reported (11). Briefly, a 96-well plate was prepared by adding the capture antibody to each well and incubate overnight. Plates were then treated with 1% bovine serum albumin (BSA) in phosphate buffered saline (PBS) for 1 h, to prevent non-specific binding. Standards and diluted serum samples were added to sample wells and incubated for 2 h at room temperature (RT), followed by incubation with the detection antibody for 2 h at RT. Afterwards, the streptavidin-conjugated to horseradish peroxidase (HRP) was added to each well and incubated at RT for 20 min previous to the addition of the substrate solution in 1:1 H_2_O_2_ and tetramethyl-benzidine to each well (20 min at RT in the dark). The reaction was interrupted by adding a stop solution (2NH_2_SO_4_) and the absorbance was measured by a spectrophotometer (FLUOStar OPTIMA, Microplate Reader, BMG, Ortenberg, Germany), using 450 nm as the primary wavelength and 570 nm as a reference wavelength. After serum leptin and ObR measurement, the FLI was calculated based on the ratio between leptin/ObR serum levels (32).

### Assessment of the Leptin and ObR Status by Immunohistochemistry (IHC)

Initially, the feline mammary carcinoma formalin fixed paraffin-embedded (FFPE) samples were stained with haematoxylin-eosin to select a representative tumor area (*n* = 58) and a normal tissue area to be used as control (*n* = 20). FFPE samples were sectioned in slices with 3 μm thickness (Microtome Leica RM135, Newcastle, UK) and mounted on a glass slide (SuperFrost Plus, Thermo Fisher Scientific, Massachusetts, USA). On PT-Link module (DAKO, Agilent, Santa Clara, USA), samples were deparaffinized, hydrated and antigen retrieval was performed for 20 min at 96°C using Tris-EDTA buffer pH 9.0 (EnVision™ Flex Target Retrieval Solution High pH, DAKO). Then, slides were cooled for 30 min at RT and immersed twice for 5 min in distilled water. IHC technique was performed with commercial solutions from the Novolink™ Max Polymer Detection System Kit (Leica Biosystems, Newcastle, UK). Before antibody incubation, tissue samples were treated with Peroxidase Block Novocastra Solution (Leica Biosystems) for 15 min and the unspecific antigenic recognition was inhibited by incubation with Protein Block Novocastra Solution (Leica Biosystems) for 10 min. Finally, tissue samples were incubated at RT for 1 h, in a humidified chamber, with the following primary antibodies: anti-leptin antibody (ab3583, Abcam, Cambridge, UK) and anti-ObR antibody (ab104403, Abcam), both diluted at 1:200. The slides were washed twice, for 5 min, between all the incubation steps, using a PBS solution at pH 7.4. Then, the detection polymer was incubated for 30 min at RT, and detection was performed using diaminobenzidine (DAB substrate buffer and DAB Chromogen, Leica Biosystems) for 5 min. Later, samples were counterstained with Gills haematoxylin (Merck, New Jersey, USA) for 5 min, dehydrated in an ethanol gradient and xylene, and mounted using Entellan mounting medium (Merck). Antibodies were predicted to react with the feline proteins, occurring in cell membrane and cytoplasm (52, 53). Human breast tissue and feline liver were used as positive controls for the leptin staining, showing a positive staining in glandular cells (25, 52), hepatocytes (54, 55), and a faint staining in cholangiocytes (56), respectively. For the ObR, human and feline kidney were used as positive controls, showing a immunostaining in tubular and some glomerular cells, as previously reported (55, 57). Sections of the feline mammary tissues analyzed were used as a negative controls.

Leptin and ObR were evaluated in the glandular epithelium of the tumor, stromal tissue and tumor infiltrating inflammatory cells. To access proteins immunoreactivity we used a previously reported scoring system (14, 25, 52) and the H-Score published by the American Society of Clinical Oncology (ASCO). The final IHC score was obtained by multiplying the positive cells (0 = absence of staining; 1 = all cells stained), by the highest staining intensity (Table 2), varying from 0 to 3, with tissue samples scored as 0 considered negative, and samples scored as 3 as highly reactive. All slides were subjected to blind scoring, by two independent and experienced pathologists.

**Table 2:** Scoring criteria of immunostaining assay for leptin and ObR.

	**Staining intensity**	
	**Score**	**Interpretation**
**Stained tumor cells (0–1)**	0	No staining
	1	Weak
	2	Moderate
	3	Strong^†^

*Final IHC score = stained tumor cells x staining intensity score (0–3)*.

*Three microscopic fields were analyzed at 400× magnification (^†^High protein reactivity)*.

### Statistical Analysis

Statistical analysis was carried out using the GraphPad Prism software, version 5.04 (California, USA), with two-tailed *p* < 0.05 considered statistically significant for a 95% confidence level (^*^p < 0.05, ^**^p < 0.01, and ^***^p < 0.001) and the average values were represented with the standard deviation.

The non-parametric Kruskal–Wallis test was performed to compare leptin and ObR results between healthy cats and cats with mammary carcinomas stratified by tumor subtype. Receiver-operating characteristic (ROC) curves were performed to choose the optimal cut-off value for serum leptin and ObR levels, and to determine the specificity and sensitivity of the technique to diagnose the disease. The non-parametric Mann-Whitney test was used to compare the serum levels of both proteins with several clinicopathological features. Survival analysis was performed using the Kaplan–Meier test to evaluate the disease-free survival (DFS) in cats with mammary carcinomas. Correlations between serum ObR levels and the previously reported serum CTLA-4, TNF-α, PD-1, and PD-L1 concentrations (10, 11) were investigated using the Spearman's rank correlation coefficient.

## Results

### Cats With Mammary Carcinoma Showed Lower Free Leptin Index

**Figure 1:**
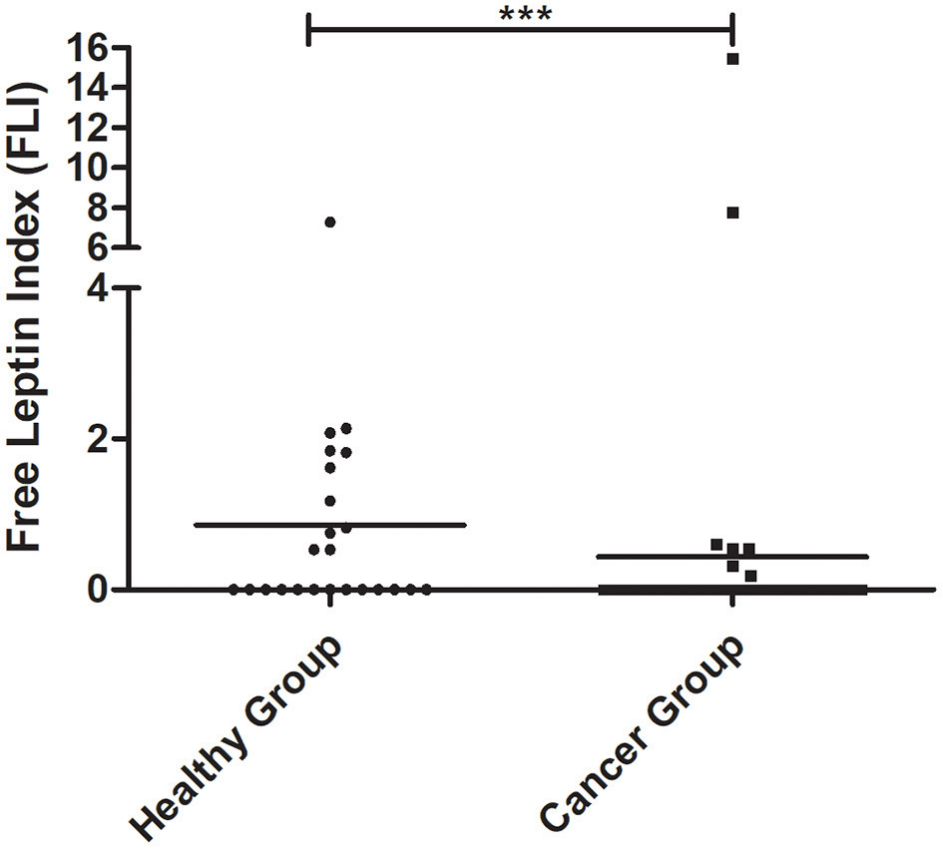
Dot plot diagram showing that the Free Leptin Index (FLI) was significantly elevated in healthy animals than in cats with mammary carcinoma (***p = 0.0006).

The Free Leptin Index (FLI) was determined in the serum samples of cats with mammary carcinomas and compared with healthy animals. Results obtained showed that cats with disease had a significantly lower FLI than the control group (0.44 vs. 0.86, *p* = 0.0006, Figure 1). Moreover, the same results were obtained if the outliers were removed from the analysis (*p* = 0.0005).

**Figure 2:**
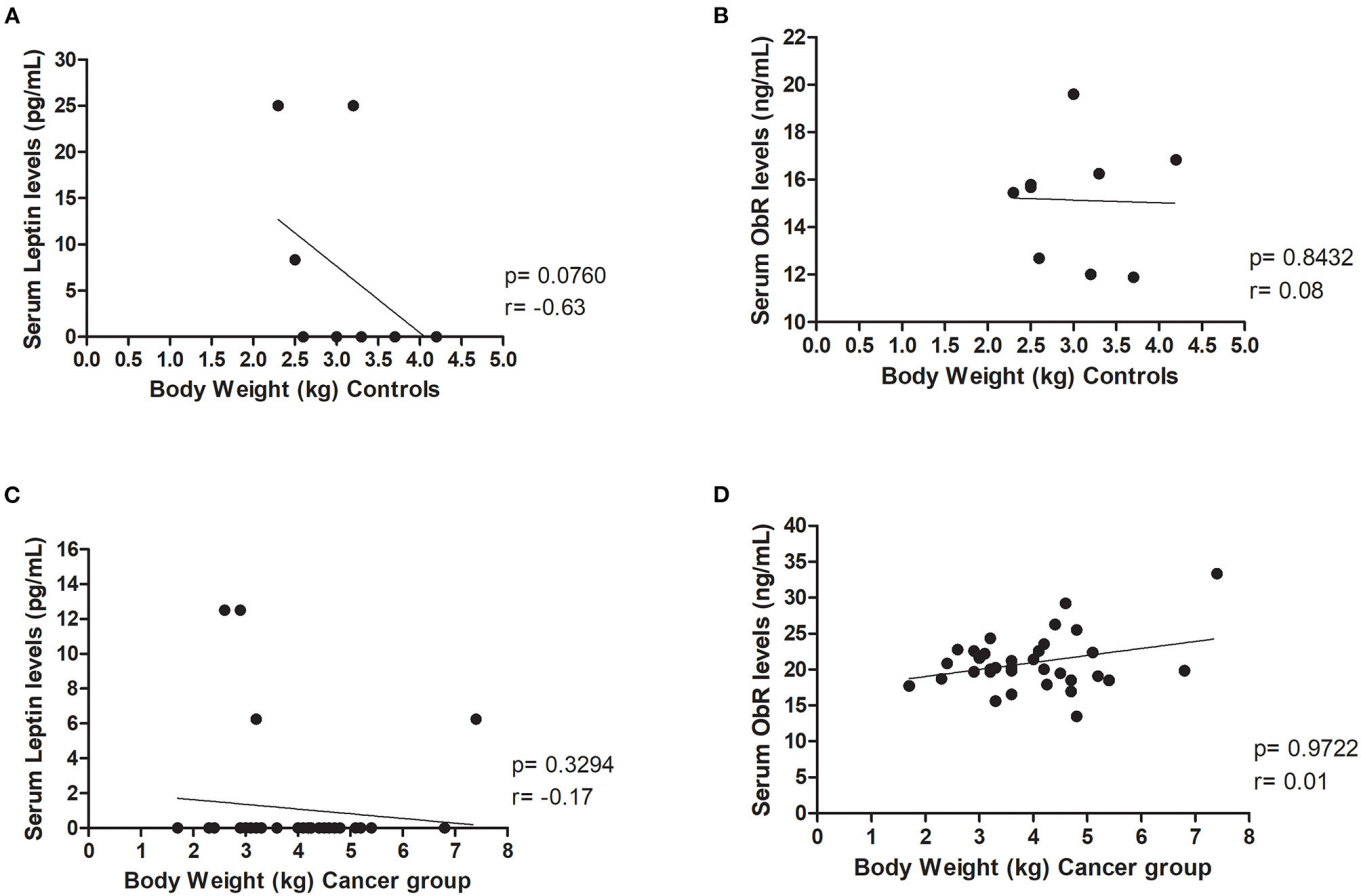
Body weight did not influence leptin, neither ObR serum levels in healthy and diseased animals. Correlations were not found between **(A)** serum leptin (*p* = 0.0760) or **(B)** ObR (*p* = 0.8432) levels and body weight in the control group. Furthermore, evaluating the cancer group, no correlations were detected between **(C)** serum leptin (*p* = 0.3294) or **(D)** ObR (*p* = 0.9722) levels and feline body weight.

In addition, results revealed that body weight did not influence serum leptin and ObR levels, both in the control group (*p* = 0.0760 and *p*= 0.8432, respectively, Figures 2A,B) and in the cancer group (*p* = 0.3294 and *p* = 0.9722, respectively, Figures 2C,D).

### Cats With Luminal B or HER2-Positive Mammary Carcinomas Showed Decreased Serum Leptin Levels

**Figure 3:**
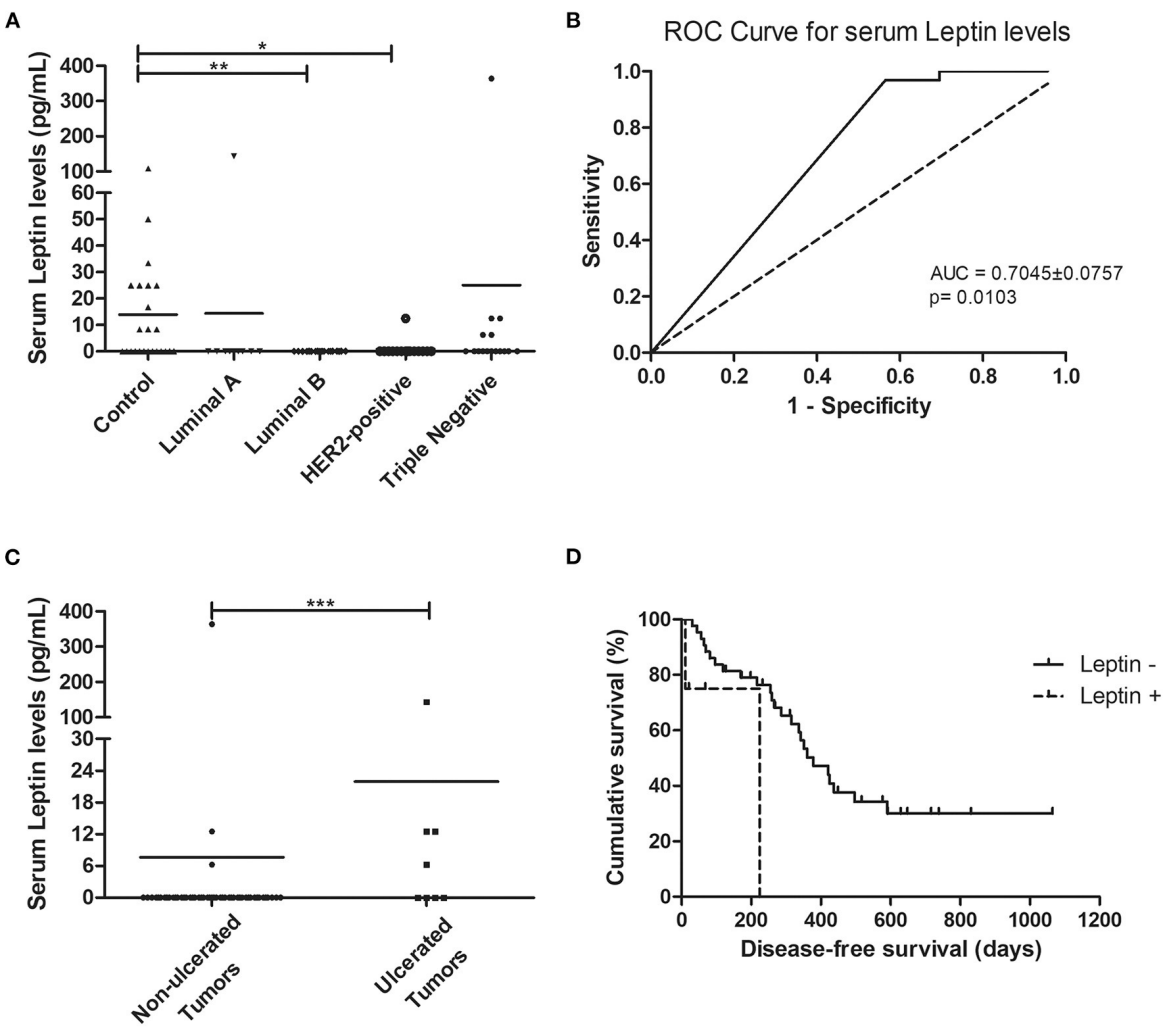
Cats with luminal B and HER2-positive mammary carcinomas showed decreased serum leptin levels, although cats with ulcerated tumors exhibited serum leptin levels above the cut-off value of 4.17 pg/mL, being associated with shorter disease-free survival. **(A)** Dot plot diagram showing the distribution of serum leptin levels (pg/mL) among healthy animals (control) and cats stratified by the mammary carcinoma subtype. Significant decreased serum levels of leptin were found in cats presenting luminal B or HER2-positive subtypes in comparison to healthy animals (*p* = 0.0025). **(B)** The optimal cut-off of serum leptin levels to predict mammary carcinoma was determined to maximize the sum of the sensitivity and specificity (4.17 pg/mL; AUC = 0.7045 ± 0.0757, 95% CI: 0.5561–0.8528, *p* = 0.0103; sensitivity = 96.9%; specificity = 43.5%). **(C)** Dot plot diagram showing that serum leptin levels were significantly higher in cats with ulcerated tumors (*p* = 0.0005). **(D)** Cats with mammary carcinoma and serum leptin levels higher than 4.17 pg/mL had a lower DFS (*p* = 0.0217). *p < 0.05; **p < 0.01; ***p < 0.001.

Regarding the serum leptin levels, results obtained showed that cats with luminal B or HER2-positive mammary carcinomas had lower serum leptin levels than healthy animals (0.00 vs. 13.89 pg/ml, *p* < 0.01; 0.83 vs. 13.89 pg/mL, *p* < 0.05, respectively, Figure 3A), and considering the analysis with no outliers, the same results could be reported (*p* = 0.0021). The optimal cut-off value to predict mammary carcinoma was 4.17 pg/ml with an area under the ROC curve (AUC) of 0.7045 ± 0.0757 (95% CI: 0.5561–0.8528, *p* = 0.0103; sensitivity = 96.9%; specificity = 43.5%; Figure 3B). Considering this analysis with no outliers, an AUC = 0.6732 ± 0.0731 (95% CI: 0.5299–0.8164, *p* = 0.0158; sensitivity = 92.9%; specificity = 43.4%) was obtained, leading to the same results. Further statistical analysis, revealed that serum leptin levels above the cut-off value were associated with tumor ulceration (*p* = 0.0005, Figure 3C; or *p* = 0.0009, if no outliers were considered) and shorter DFS (117 vs. 314 days, *p* = 0.0217, Figure 3D; or *p* = 0.0245, if the outliers were removed).

### Cats With Mammary Carcinoma Showed Elevated Serum Levels of ObR and of Inflammation Mediators

**Figure 4:**
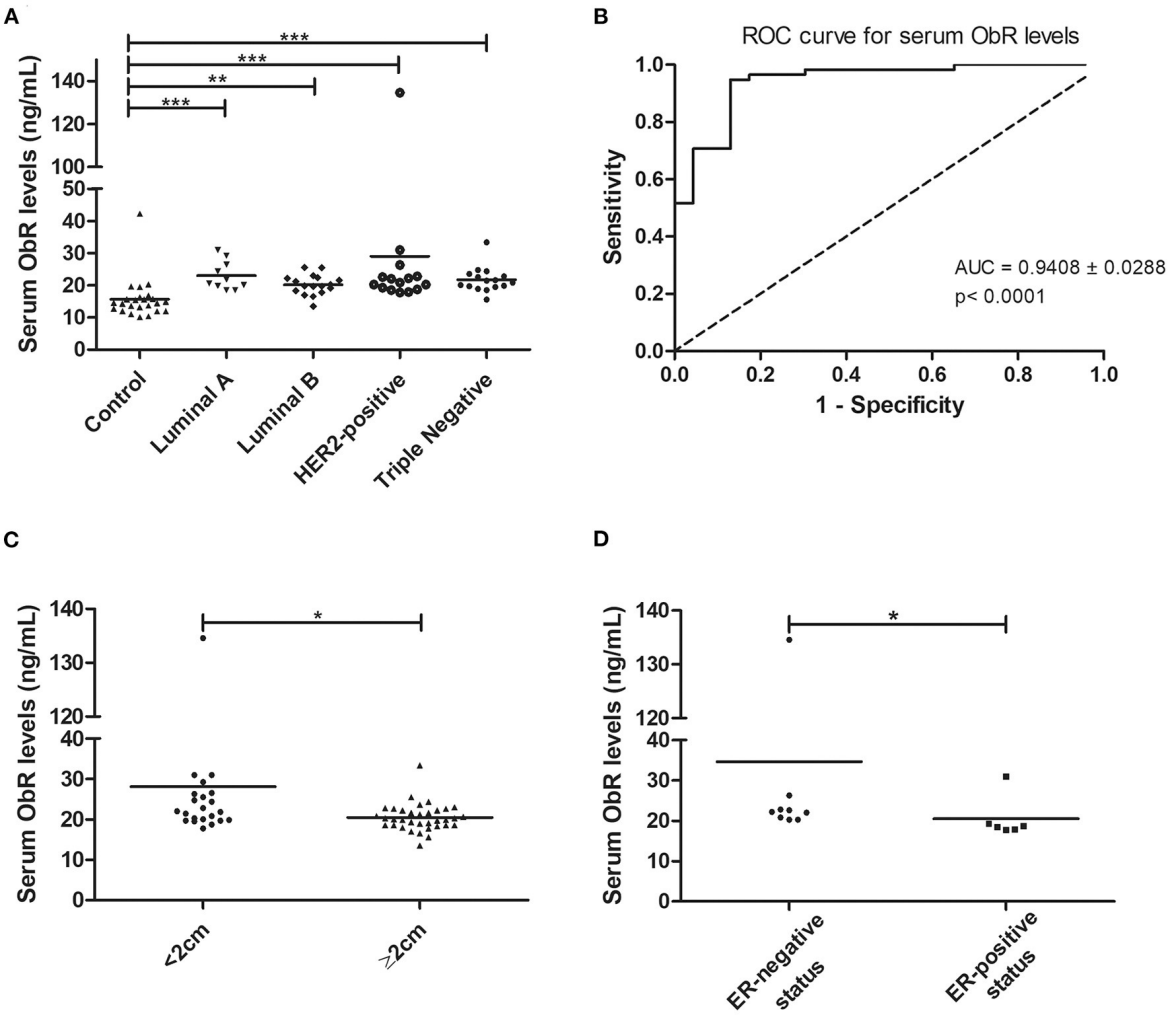
Cats with mammary carcinoma showed elevated serum ObR levels, with serum concentrations above 16.89 ng/mL being associated with smaller tumors and an ER-negative status. **(A)** Dot plot diagram showing the distribution of serum ObR levels (ng/mL) in heathy animals (control) and in cats with mammary carcinoma stratified by the molecular subtype. Significant higher serum levels of ObR were found in all tumor subtypes in comparison to healthy animals (*p* < 0.0001). **(B)** The optimal cut-off value of serum ObR levels to predict cats with mammary carcinoma was 16.89 ng/mL with an AUC of 0.9408 ± 0.0288 (95% CI: 0.8842–0.9973, *p* < 0.0001; sensitivity = 94.8%; specificity = 87.0%). **(C)** Dot plot diagram showing that serum ObR concentrations were significantly low in tumors larger than 2 cm (*p* = 0.0118). **(D)** Dot plot diagram displaying a positive association between higher serum ObR levels and ER-negative status (*p* = 0.0291). *p < 0.05; **p < 0.01; ***p < 0.001.

Considering the above results, the serum ObR levels were also evaluated. When the animals were grouped according to the tumor subtype, a significant difference was found between the mean ranks of at least one pair of groups (*p* < 0.0001, with or without outliers). Results revealed that serum ObR levels were significantly higher in animals with mammary carcinoma than in controls, independently of molecular subtype (control group 15.67 ng/ml; luminal A 23.04 ng/ml, *p* < 0.0001; luminal B 20.18 ng/ml, *p* < 0.001; HER2-positive 28.99 ng/ml, *p* < 0.0001; triple-negative 21.70 ng/ml, *p* < 0.0001; Figure 4A). Furthermore, the optimal cut-off value calculated for cats with mammary carcinoma was 16.89 ng/ml, with an AUC of 0.9408 ± 0.0288 (95% CI: 0.8842–0.9973, *p* < 0.0001; sensitivity = 94.8%; specificity = 87.0%; Figure 4B). If the outliers were removed from the analysis, the same results were obtained, with an AUC = 0.9397 ± 0.0293 (95% CI: 0.8823–0.9972, *p* < 0.0001; sensitivity = 94.7%; specificity = 87.0%).

**Figure 5:**
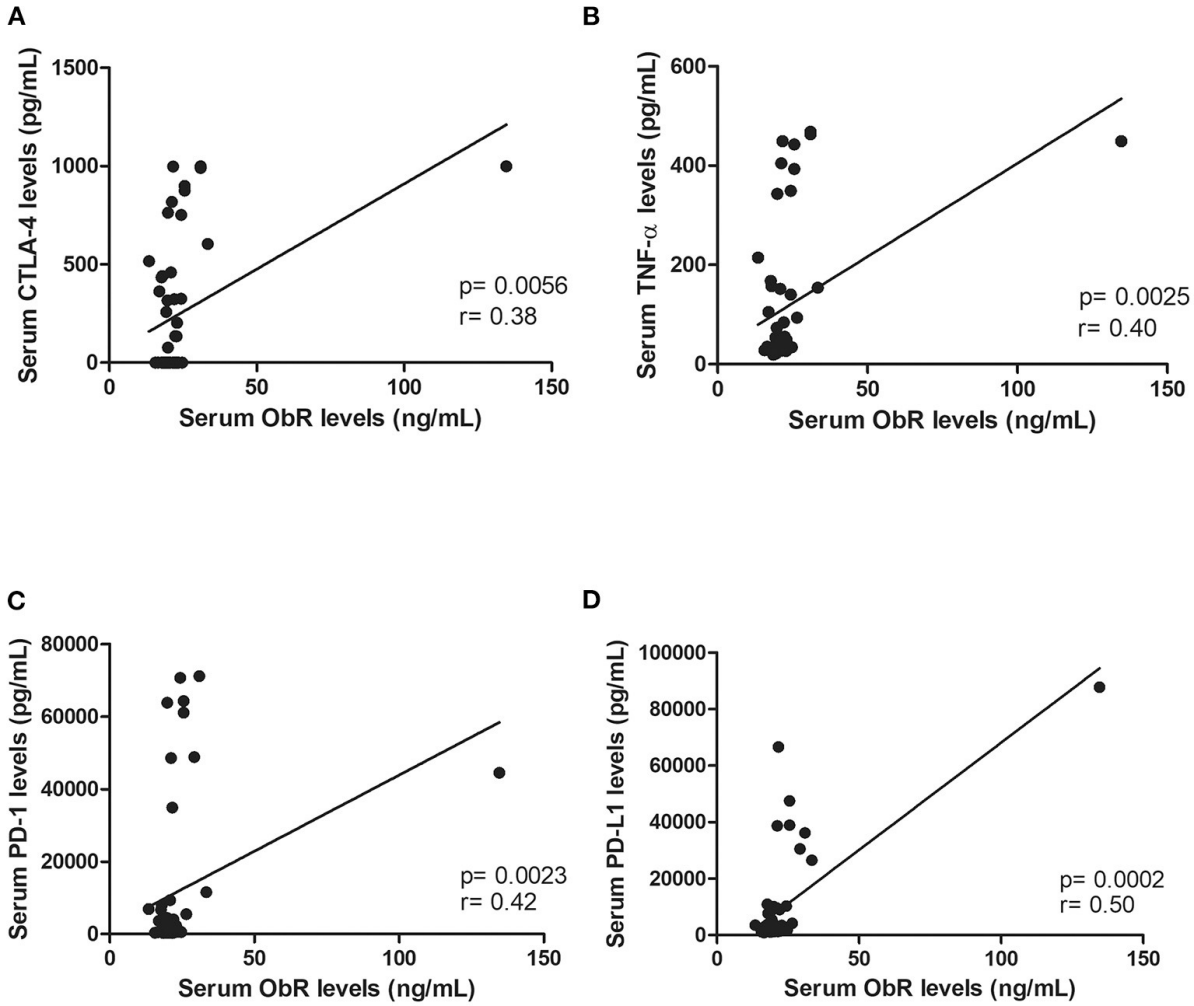
Serum ObR levels showed a positive correlation with inflammatory mediators, namely **(A)** serum CTLA-4 levels (*p* = 0.0056), **(B)** serum TNF-α levels (*p* = 0.0025), **(C)** serum PD-1 levels (*p* = 0.0023), and **(D)** serum PD-L1 levels (*p* = 0.0002).

In addition, elevated serum ObR levels were associated with smaller tumors (*p* = 0.0118, Figure 4C; *p* = 0.0248, if no outliers were considered) and with cats had an ER-negative status (*p* = 0.0291, Figure 4D; *p* = 0.0452, if no outliers were considered). Finally, a positive correlation was found between serum ObR levels and serum levels of CTLA-4 (*r* = 0.38, *p* = 0.0056, Figure 5A), TNF-α (*r* = 0.40, *p* = 0.0025, Figure 5B), PD-1 (*r* = 0.42, *p* = 0.0023, Figure 5C), and PD-L1 (*r* = 0.50, *p* = 0.0002, Figure 5D). Removing the outliers from our data, the same results could be reported (CTLA-4: *r* = 0.34, *p* = 0.0153; TNF-α; *r* = 0.37, *p* = 0.0064; PD-1: *r* = 0.39, *p* = 0.0002; and PD-L1: *r* = 0.47, *p* = 0.0007).

### Leptin and ObR Are Overexpressed in Luminal B and Triple-Negative Mammary Carcinomas

**Figure 6:**
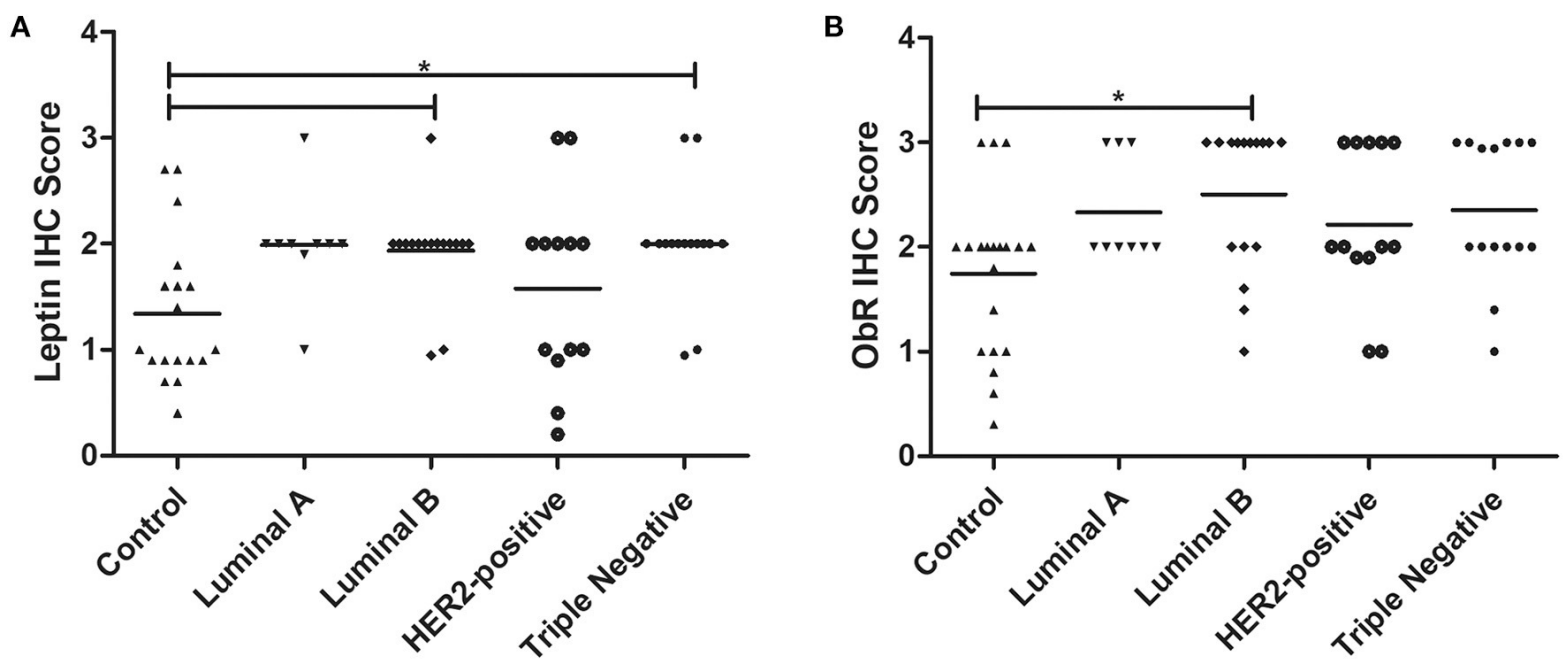
Final IHC scores for leptin **(A)** and ObR **(B)** in cats with mammary carcinoma stratified by the tumor subtype and compared with controls. **(A)** Leptin expression was significantly higher in luminal B and triple-negative subtypes (*p* = 0.0046). **(B)** Expression of ObR was statistically higher in luminal B tumor subtype (*p* = 0.0425).

**Figure 7:**
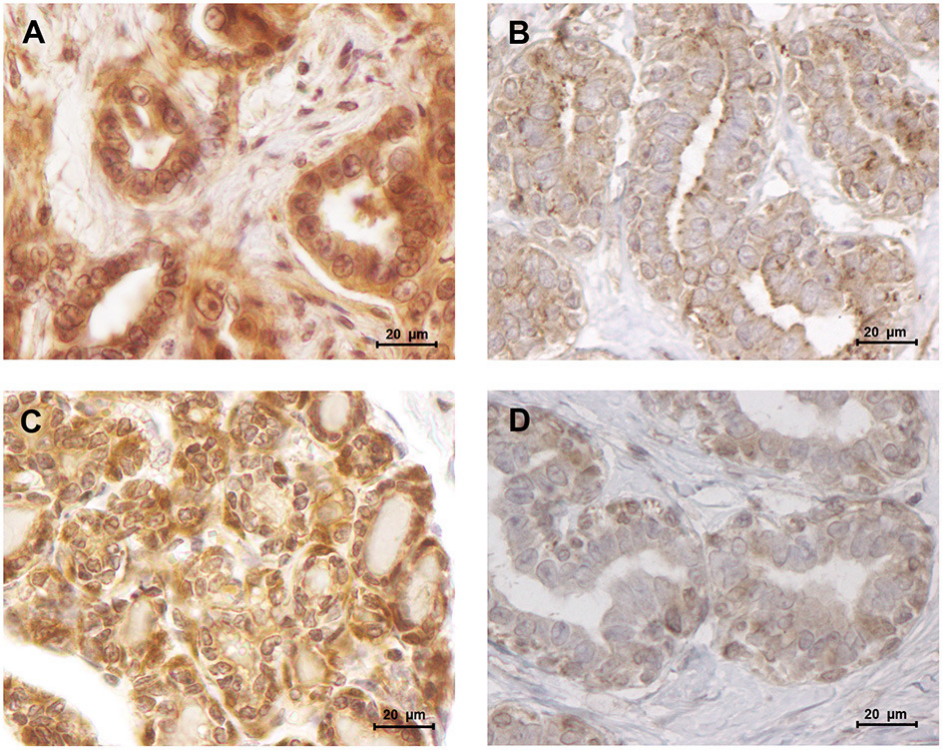
Leptin and ObR were overexpressed in luminal B mammary carcinomas. **(A)** Leptin overexpression in a luminal B mammary carcinoma (IHC score of 1.93) contrasting with **(B)** a low staining intensity detected in normal mammary tissues (IHC score of 1.34). **(C)** Luminal B mammary tumors showed a higher staining intensity for ObR (IHC score of 2.50), **(D)** than normal mammary tissues (IHC score of 1.75) (400× magnification).

The obtained results revealed that cats with luminal B or triple-negative mammary carcinoma showed a higher leptin IHC score in the tumor glandular cells, comparing to the healthy control samples (1.93 vs. 1.34, *p* < 0.05; 2.00 vs. 1.34, *p* < 0.05, respectively; Figures 6A, 7A,B). Regarding the leptin receptor, the IHC score was also significantly higher in animals with a luminal B tumor subtype than in healthy animals (2.50 vs. 1.75; *p* = 0.0425; Figures 6B, 7C,D).

**Figure 8:**
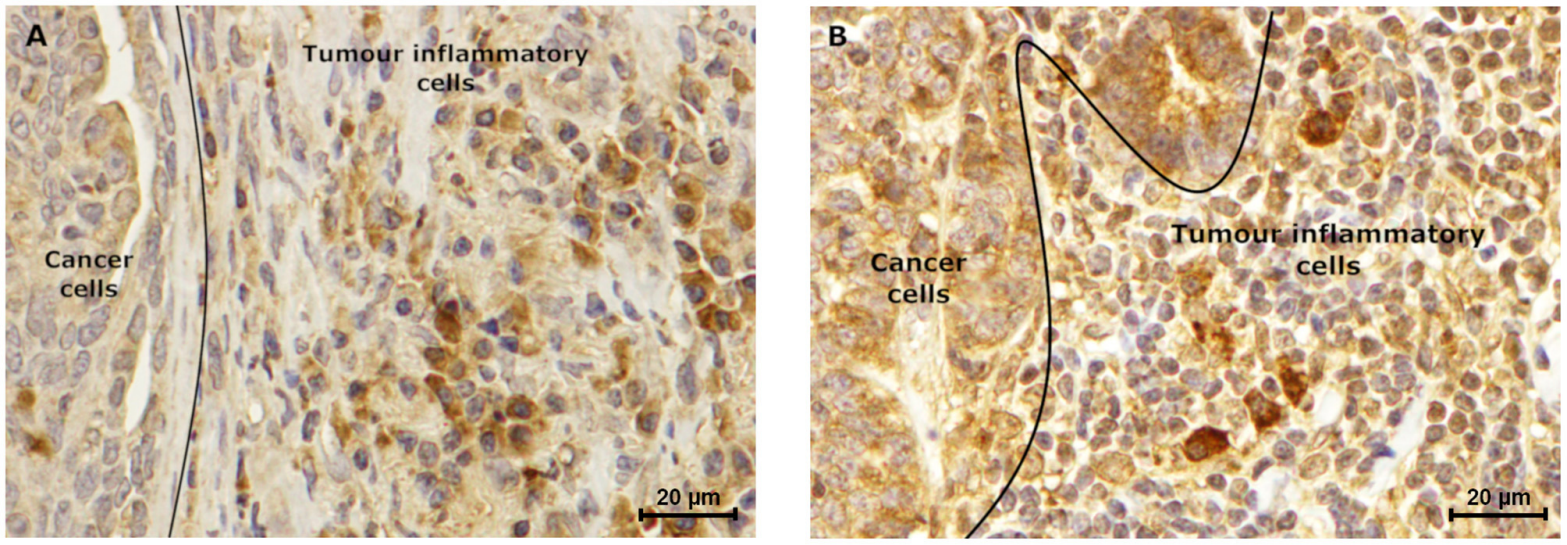
Tumor inflammatory cells express leptin and ObR. Luminal B carcinoma subtype showed a **(A)** positive leptin staining (IHC score of 1.5), which is lower when compared to the **(B)** ObR immunostaining (IHC score of 2.5) of tumor inflammatory cells. Furthermore, in both samples higher staining intensity was observed in macrophages, when compared to lymphoid cells (IHC score of 2.0 vs. 1.2, respectively for leptin, and IHC score of 3.0 vs. 2.0, respectively for ObR) (400× magnification).

Furthermore, the immunostaining reveals to be positive in the stroma cells, in 72.2 and 18.2% of the tumors, for leptin (IHC score of 0.79) and ObR (IHC score of 1.1), respectively. Moreover, was observed that, independently of the tumor subtype, a mean of 81 ± 2.5% of the tumor inflammatory mononuclear cells presented to be positive for the leptin staining (mean IHC score of 2.0 for the macrophages and mean IHC score of 1.6 for the lymphoid cells, Figure 8A). The same analysis, considering the ObR revealed a positive staining for a mean of 87.6 ± 2.5% of the tumor inflammatory mononuclear cells (mean IHC score of 2.62 for the macrophages and mean IHC score of 1.33 for the lymphoid cells, Figure 8B).

**Figure 9:**
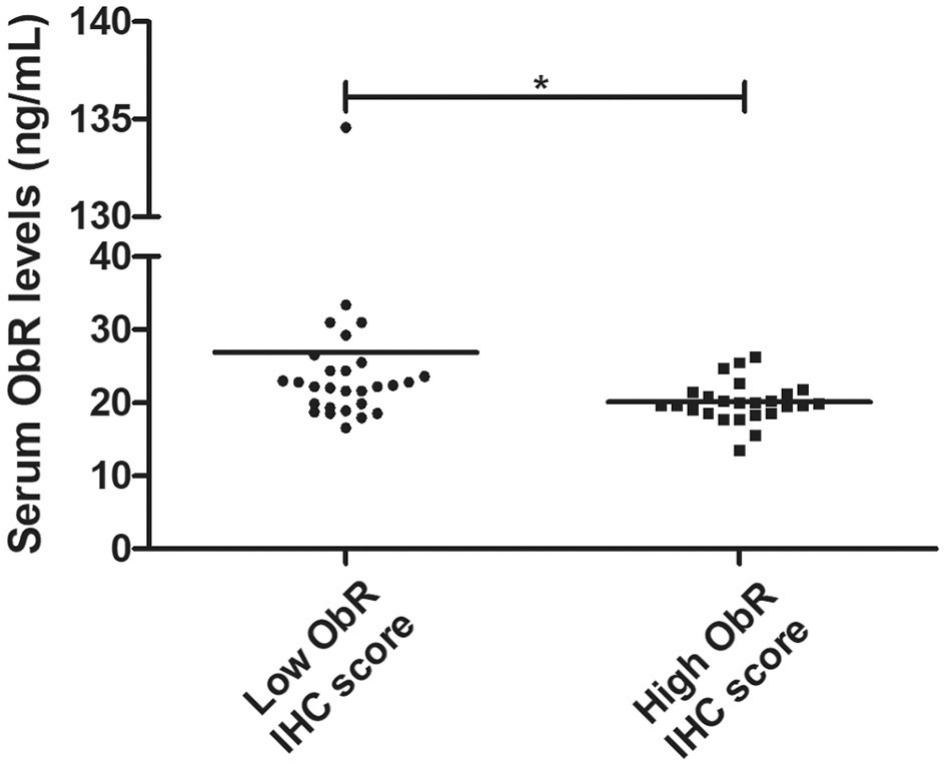
Dot plot diagram showing a negative correlation between serum ObR levels and tumor ObR IHC score (*p = 0.0103).

In addition, our findings revealed that serum ObR levels are negatively correlated with the ObR IHC score, with cats presenting higher serum ObR levels showing mammary tumors with lower ObR IHC scores (*p* = 0.0103, Figure 9; *p* = 0.0244 if the outliers were removed from the analysis).

## Discussion

Although spontaneous FMC has been proposed as a suitable model for human breast cancer studies, the role of the leptin/ObR axis has never been evaluated in cats. In humans, previous studies showed that leptin and ObR overexpression are associated with pro-inflammatory and pro-tumorigenic effects, particularly in overweight women (13, 15). Moreover, some studies reported increased serum leptin levels with aging (58, 59). In this study, the healthy group presented a mean age lower than the tumor group, and despite what is reported in human and rats (58, 59), the results obtained demonstrated that cats with mammary carcinoma have a reduced Free Leptin Index (FLI) in comparison to the healthy group (*p* = 0.0006), not only due to the increase in serum ObR levels (60), but also suggesting that diseased animals may have decreased soluble leptin levels, as reported in pre-menopausal women with breast cancer (61) and colon cancer patients (62). These results indicate that serum leptin may be recruited by mammary cancer cells to promote tumor growth and cell migration (23). Indeed, cats with luminal B or HER2-positive mammary carcinoma showed significantly lower serum leptin levels when compared with controls (*p* < 0.001 and *p* < 0.05, respectively), revealing that serum leptin levels are downregulated in tumors with PR-positive status (14) and/or HER2-positive status (27). In contrast, cats with luminal A showed elevated serum leptin levels, indicating that ER overexpression in the tumor may promotes leptin expression (14). Regarding the elevated serum leptin levels found in cats with triple-negative mammary carcinomas, studies demonstrated that leptin induces cell proliferative capacity (e.g., via Wnt/β-catenin pathway) (22, 43) and promotes cell survival by interacting with Bcl-2 proteins, being associated with more aggressive tumors (63). Indeed, our results revealed that elevated serum leptin levels occur in an advanced stage of the disease, being significantly associated with tumor ulceration (*p* = 0.0005) and shorter DFS (*p* = 0.0217), as reported for women with breast cancer (14, 15).

In parallel, as documented in breast cancer patients (14, 64), all cats with mammary carcinoma showed higher serum ObR levels than healthy controls (*p* < 0.0001). Also higher serum ObR levels were correlated with smaller tumor size (*p* = 0.0118), suggesting that ObR shedding occurs in small tumors, modulating the serum levels of free leptin (28). Moreover, our results further support the hypothesis that malignant cells in larger tumors maintain the ObR expression on its surface to increase their survival and growth (19). Interestingly, the higher serum ObR levels were found in cats with mammary carcinomas presenting a HER2-positive/ER-negative status (*p* = 0.0291), as reported for human breast cancer patients (14), confirming the crosstalk between the leptin/ObR axis and the EGFR downstream signaling pathway (65).

In addition, this study discloses the utility of leptin and ObR as promising diagnostic biomarkers to differentiate animals with FMC from healthy cats (cut-off value of 4.17 pg/mL for leptin and 16.89 ng/mL for ObR).

We also found that serum ObR levels were positively correlated with serum CTLA-4 (*p* = 0.0056), TNF-α (*p* = 0.0025), PD-1 (*p* = 0.0023) levels as reported in breast cancer patients (39), and with serum PD-L1 levels (*p* = 0.0002). Indeed, previous studies showed that activation of the leptin/ObR axis can result in a chronic inflammatory status (35, 66), a well-known risk factor for breast cancer, with leptin being involved in CD4+ T-regulatory cells differentiation due to ObR overexpression on lymphocyte plasm membrane (67). These activated CD4+ T-regulatory cells express CTLA-4(35) and PD-1, two immune-inhibitory checkpoint molecules that downregulate T-cell immune responses (37), leading to tumor development (68), and contributing to cell growth (69). On the other hand, in an attempt to control the tumorigenesis process, CD4+ T-regulatory cells secrete TNF-α (38), a molecule that shows a dual role in immunomodulation, being also expressed by cancer cells (70), acting as an autocrine growth factor (71). Altogether, these findings provide support for the crosstalk between the leptin/ObR axis and tumor immunoediting mechanisms, contributing to an immunosuppressive status in cats with mammary carcinoma (10, 11).

The immunostaining analysis of the tumor and normal tissue samples revealed that luminal B and triple-negative mammary carcinoma subtypes (*p* < 0.05) showed leptin overexpression. Although a strong ObR expression was only detected in luminal B mammary carcinomas (*p* = 0.0425), as described in human breast cancer (25). Furthermore, several studies suggest that leptin and ObR are overexpressed in tumor tissues, due to hypoxia and/or as a response to insulin, IgF-1 and/or to estradiol (64, 72). In addition, the higher IHC scores for leptin found in luminal B carcinomas also support the previously reported association between the expression of this adipocytokine and aromatase expression, an enzyme that catalyzes the conversion of androgen into estrogen to promote tumor development via an ER-dependent mechanism (14). The overexpression of leptin detected in triple-negative mammary carcinomas is also in concordance with previous results in triple-negative breast cancer, where leptin signaling is crucial for tumor growth (29, 63), being associated with ERK and Akt pathways, both involved in breast cancer cells proliferation (23). Furthermore, the tumor inflammatory mononuclear cells revealed to be positive for leptin and ObR immunostaining, with a higher proteins expression in macrophages. In fact, leptin/ObR axis are reported as activating the inflammatory response (66, 73). Finally, our results demonstrated that cats with low ObR-expressing mammary tumors had higher serum ObR levels, indicating a negative feedback between tumor microenvironment and serum, probably due to a shedding mechanism that leads to a reduction of serum leptin levels (23, 60). Furthermore, the data obtained emphasizes the possibility of blocking the leptin/leptin receptor axis, as an adjuvant therapy in cats with luminal B and triple-negative mammary carcinoma subtypes, as reported for breast cancer patients (42, 44–46).

In conclusion, our data provide a rationale use for leptin/ObR as diagnostic and prognostic biomarkers. Indeed, cats with mammary carcinoma showed a decreased FLI, coupled with decreased serum leptin levels in animals with luminal B or triple-negative mammary carcinoma subtypes. A significant increase in serum ObR levels, was detected in all samples, independently of the tumor subtype, being associated to an immunosuppressive status. Altogether, our data indicate that cats presenting luminal B and triple-negative tumors could benefit from adjuvant therapies targeting leptin, and support the utility of spontaneous FMC as a model for comparative oncology.

## Data Availability Statement

The raw data supporting the conclusions of this article will be made available by the authors, without undue reservation.

## Ethics Statement

The animal study was reviewed and approved by CIISA - Faculdade de Medicina Veterinária. Written informed consent was obtained from the owners for the participation of their animals in this study.

## Author Contributions

AG and FF: designed the research. AG, CN, ACU, JC, and FF: performed the research. AG, CN, and FF: analyzed the data. AG and FF: wrote the paper. All authors have read and agreed to the published version of the manuscript.

## Conflict of Interest

The authors declare that the research was conducted in the absence of any commercial or financial relationships that could be construed as a potential conflict of interest.
